# Homogeneity Characterization of Textile-Integrated Wearable Sensors based on Impedance Spectroscopy

**DOI:** 10.3390/s22176530

**Published:** 2022-08-30

**Authors:** Hanen Nouri, Dhivakar Rajendran, Rajarajan Ramalingame, Olfa Kanoun

**Affiliations:** Measurement and Sensor Technology, Chemnitz University of Technology, 09126 Chemnitz, Germany

**Keywords:** multiwalled carbon nanotubes, textile sensors, pressure sensor, wearable sensors, impedance spectroscopy, constant phase element

## Abstract

One of the main challenges during the integration of a carbon/polymer-based nanocomposite sensor on textile substrates is the fabrication of a homogeneous surface of the nanocomposite-based thin films, which play a major role in the reproducibility of the sensor. Characterizations are therefore required in every fabrication step to control the quality of the material preparation, deposition, and curing. As a result, microcharacterization methods are more suitable for laboratory investigations, and electrical methods can be easily implemented for in situ characterization within the manufacturing process. In this paper, several textile-based pressure sensors are fabricated at an optimized concentration of 0.3 wt.% of multiwalledcarbon nanotubes (MWCNTs) composite material in PDMS. We propose to use impedance spectroscopy for the characterization of both of the resistive behavior and capacitive behavior of the sensor at several frequencies and under different loads from 50 g to 500 g. The impedance spectra are fitted to a model composed of a resistance in series with a parallel combination of resistance and a constant phase element (CPE). The results show that the printing parameters strongly influence the impedance behavior under different loads. The deviation of the model parameter α of the *CPE* from the value 1 is strongly dependent on the nonhomogeneity of the sensor. Based on an impedance spectrum measurement followed by parameter extraction, the parameter α can be determined to realize a novel method for homogeneity characterization and in-line quality control of textile-integrated wearable sensors during the manufacturing process.

## 1. Introduction

Textile-integrated and wearable nanocomposite pressure sensors have captured immense interest in recent years due to their high potential for use in several applications related to wearable electronic devices [1,2,3], human–machine interfaces [4,5], and health monitoring [6,7,8,9]. Textile materials generally consist of a network of fibers, such as cotton, wool, and silk, or synthetic fibers such as nylon and polyester. Textiles have many unique properties of textile materials, including flexibility, strength, durability, a light weight, and water absorption [10]. These unique properties lead to many advantages of textile-based substrates comparedto other substrate materials such as thermal permeation, a high breathability [11], a light weight, washability, biocompatibility, and comfortability [12]. However, fabricating sensors on fabric substrates directly is still challenging. Textile-based sensors often require an additional lamination or interlayer as the surfaces of textiles are generally rough, leading to weak adhesion, the delamination of desired materials, and the degradation of electrical properties. On the other hand, textile-based sensors require excellent mechanical properties to withstand bending, stretching, and twisting in the normal course of daily life. Several important factors must be considered when designing wearable sensors, including excellent flexibility and stretchability to integrate with any curved surface in a conformable and efficient manner.

As a polymer, polydimethylsiloxane(PDMS) offers excellent elasticity, a large Poisson’s ratio (0.48), and easy fabrication can be adopted. It offers a flexible, pliable, thermally stable, and chemically stable silicone rubber widely used as a soft and stretchable material in flexible sensors. However, due to its poor conductivity, it is a pure material unsuitable for sensor applications. Thus, different conductive materials, such as carbon nanomaterials (e.g., graphene and carbon nanotubes), have been introduced into PDMS to realize sensors. Already during the material fabrication, it is a big challenge to maintain the homogeneity of the resulting composite due to the hydrophobicity of the PDMS. During preparation and deposition, nanoparticles can reagglomerate, bubbles can be formed, and nanoparticle sedimentation can occur. During deposition by screen printing, the nonhomogeneity increases due to the uneven surface of the textile. The highly flexible soft elastomer used in the dielectric layer of these sensors makes them highly appropriate for measuring small forces with a high sensitivity.

Aiming to bring the sensor to mass production, there is a considerable need for sensor characterization, especially as a last step in the production process for quality control. In this work, the potential of impedance spectroscopy is investigated to characterize flexible textile-based pressure sensors. In Section 2, state-of-the-art characterization methods for nanocomposites are evaluated and compared. Section 3 illustrates nanocomposite sensors’ impedimetric behavior (resistive, capacitive, and impedance behavior). Section 4 illustrates the adequate proportion of materials and sensor design. The impedance for different samples is determined to find the adequate equivalent circuit. In Section 5, the impedance response of the sensor with different loads is shown. Later, the equivalent circuit parameters are used to study manufacturing anomalies.

## 2. Characterization Methods for Nanocomposite Sensors

Characterization methods for nanocomposite films can be divided into microcharacterization and electrical-based methods, as shown in Table 1. Examples of microcharacterization methods are scanning electron microscopy (SEM), Raman spectroscopy, atomic force microscopy (AFM), and transmission electron microscopy (TEM). Ko et al. 2021 introduced a wide range of highly sensitive, stable, reversible, and biocompatible pressure sensors based on a porous material [13]. The sensor was fabricated using carbonized cotton fabric (CCF) and thermoplastic polyurethane (TPU) composite with 7 wt.% of TPU. The sensor was characterized by scanning electron microscopy (SEM). SEM produces sensor images by scanning its surface using a focused beam of electrons. SEM results showed that the carbonized fabric fibers became finer than those in the pristine cotton fabric, indicating that the carbon-based materials remained after the carbonization process. The SEM method has a very high resolution, providing highly magnified images of backscattered electrons from sample surfaces. It is, therefore, able to provide information about the state of the nanofillers. Nevertheless, the sample should be small enough to fit inside a vacuum chamber, where the measurements can be carried out. For samples with low conductivity, a special conductive layer must be deposited on the sample before SEM imaging. Raman spectroscopy determines the vibrational energy modes of samples with the help of scattered light. In [14], a 3D piezoresistive sensor based on a nonwoven substrate and 0.1 wt.% of MWCNTs was designed and characterized by Raman spectroscopy. The results showed peaks of MWCNT at 1326, 1580, and 2644 cm${}^{-1}$, where the fiber surface was entirely covered with MWCNTs after sonication. This method had a very small scattering cross section (about 10–30 cm${}^{2}$) and needed a high laser intensity to obtain a detectable signal. It had, therefore, difficulties in measuring low concentrations of molecules on samples, and the sample was heated by the laser radiation, which could destroy it. It was unsuitable for characterizing the homogeneity of the whole surface of a macrosensor and was difficult to implement in situ for the mass production of sensors. In [15], atomic force microscopy (AFM) was used to characterize an electrochemical flexible biosensor. AFM is a very high resolution type of scanning probe microscopy, and its resolution reaches fractions of a nanometer. The sensor was fabricated using Gold (Au), molybdenum disulfide nanoparticles ( Mo$S_{2}$ NPs), and Au (Au/Mo$S_{2}$/Au nanolayer) on the polyethylene terephthalate (PET) substrate. The AFM results of the PET substrate indicated a height of 1.296 nm. When Au was sputter-coated on the PET substrate, the height of the immobilized Mo$S_{2}$ NPs was about 155.81 nm. The AFM is an excellent method to study the roughness of surfaces. However, it can scan only a single nanosized surface at a time and the sample can be damaged during detection. In [16], a PDMS/MWCNT capacitive pressure sensor using different concentration of MWCNTs (1, 2, 4, 5, and 6 wt.%) was investigated using transmission electron microscopy (TEM). This characterization method uses a beam of electrons transmitted through a specimen to form an image. Results showed that the MWCNT nanofillers were well-dispersed at lower concentrations of MWCNT, whereas at higher doping, few agglomerates were found. Based on the distribution of MWCNTs in the PDMS matrix, it could be confirmed that the percolation phenomenon occurred at a 4 wt.% doping concentration. The high-resolution transmission electron spectroscopy (HR-TEM) photomicrographs also found that the average diameter of MWCNTs in nanocomposite was around 20 nm. Using this method required a laborious sample preparation, which destroyed the original sample. Microcharacterization is generally performed under laboratory conditions. Only a sample or even a small surface of a sample can be tested for quality control. These methods can provide interesting information about nanofiller concentration, and the cohesion between the polymer and the nanofillers, but they are destructive, time-consuming, and limited. They are therefore not suitable for integration in a fully automatic characterization step in-line during the production.

On the other hand, electrical-based methods such as resistance, capacitance, and impedance measurements can be used to characterize the fabrication method’s efficiency in measuring the electromechanical performance of the sensors. In [17], a flexible pressure sensor was fabricated using PDMS/MWCNTs at 0.1 wt.% and characterized by resistance measurements. The results showed that the resistance dropped drastically as the pressure increased to 7 mmHg, gradually decreasing to 20 mmHg. The van der Pauw method is a four-point resistivity measurement technique and is commonly used for resistivity measurement, sheet resistance, and the Hall coefficients of sensitive films. In [18], the van der Pauw method was applied to optimize the fabrication method, electrode size, and electrode shape of graphene oxide (GO)-based cotton electrodes for electrocardiography (ECG) application. However, the van der Pauw method has many disadvantages as it needs an absolute calibration of the nanovoltmeter, a good contact to reduce the influence of the contact resistance, and for every condition, many measurements should be carried out. Furthermore, this method can not provide information about the capacitive behavior of the sensor. In other works [19], capacitive measurements were used to characterize capacitive sensors printed on a flexible textile substrate. The resistance and capacitance techniques have been implemented mainly separately and have not yet been comprehensively implemented to provide information about the complex conduction mechanisms of nanocomposites [20,21]. In [22], a piezoresistive strain sensor was characterized using impedance spectroscopy. The equivalent circuit of the impedance spectra was a parallel combination of resistance in parallel with a constant phase element (CPE). The sensor showed frequency-independent and frequency-dependent characteristics under the applied frequency. Impedance spectroscopy is attractive as a real-time and nondestructive characterization technique [23]. It is a frequency-dependent method that provides information about the change in piezoresistivity, the conduction mechanism of the sensor, its reproducibility, and the polymer and the complex network of the MWCNTs [21]. It can be integrated easily as the last step in the production process for quality control.

## 3. Impedimetric Behavior of Nanocomposite Films

### 3.1. Resistive Behavior

**Figure 1:**
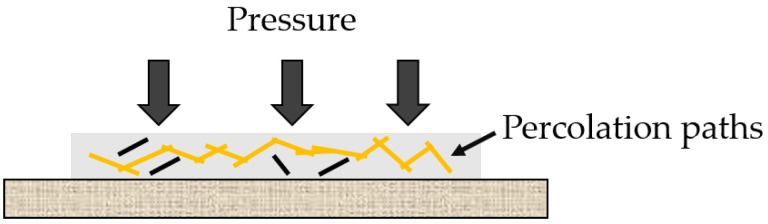
Principle of nanocomposite-based pressure sensors.

MWCNTs have a high electric conductivity, which overcomes that of polymers. At the percolation range, electrical conductive paths begin to form. This is why the electrical conductivity changes by several orders of magnitude when the MWCNTs concentration changes or the nanocomposite is subjected to pressure. The polymer matrix changes its electrical behavior from an insulator to a conductor (Figure 1). This equation can describe this phenomenon:

(1) $$\sigma =\sigma_{0}{\left(\rho -\rho_{c}\right)}^{t}for\rho >\rho_{c}$$

where $\sigma_{0}$ represents the physical parameter related to the intrinsic conductivity of MWCNTs, and *t* represents the critical exponent whose influence is governed by the system’s dimensionality. According to percolation theory, the electrical conductivity increases abruptly at a critical concentration of MWCNTs called the percolation threshold ($\rho_{c}$). Two types of resistance contribute to the resistance of a nanocomposite: the intrinsic resistance $R_{tube}$ of the MWCNTs and the intertube resistance $R_{junction}$. The intertube resistance can be either the contact resistance $R_{C}$ of MWCNTs in physical contact, e.g., ohmic contact to a metallic electrode, or the tunneling resistance $R_{T}$ of MWCNTs separated by a small gap, e.g., polymer, or a combination of both resistances as follows:

(2) $$R\left(P\right)=R_{tube}+R_{junction}$$

(3) $$R_{T}=\frac{h^{2}d}{Ae^{2\sqrt{2m\lambda }}}xe^{\frac{4d\pi }{h}\sqrt{2m\lambda }}$$

where *d* is the distance between MWCNTs, *h* is the Planck constant, *e* is the quantum of electricity, λ is the barrier height of energy, *m* is the electron mass, and *A* is the cross-sectional area of the tunnel [24].

Several parameters influence the overall resistance, including the properties of the polymer, the tunneling gap D between adjacent nanotubes, and the nanotubes’ electronic properties. Beginning at low concentrations of MWCNTs and increasing their quantity, the electrical conductivity increases in a nonlinear way. At a middle MWCNTs concentration, conductive paths begin to be built in the percolation range, allowing the current to flow through the MWCNTs, which are more in contact. As the MWCNTs concentrations exceed the percolation threshold, the conductive filler network closely resembles the classical geometrical percolation theory. At a high MWCNT concentration, the resistance sensitivity becomes low since the MWCNTs are increasingly integrated into the polymer, and the conduction occurs through quantum tunneling, in which the tunneling resistance magnitude is greater than that of direct MWCNTs contacts.

Figure 1 illustrates the nanocomposite pressure sensor’s principle under the influence of a compression and relaxation behavior. Applying pressure to the polymer nanocomposite leads to a change in the distance between MWCNTs. Since the MWCNTs are embedded in the polymer, there is always a minimum distance between the dispersed MWCNTs. Therefore, tunneling takes place between the MWCNTs. Under pressure, the gap between the MWCNTs narrows, favoring further tunneling between adjacent MWCNTs and the overall resistance of the sensor decreases.

### 3.2. Capacitive Behavior

Polymer-carbon nanocomposite capacitive sensors can be divided into two categories based on their electrode structures. They can have the structure of a parallel plate capacitor consisting of two electrodes separated by the nanocomposite sensor material. Another structure consists of interdigitated electrodes applied under the sensing layer. Although both structures can measure the sensor response as a change in capacitance under pressure, interdigitated electrodes are more cost-effective, do not change their geometry under pressure, and are able to detect a wide range of pressures [25]. Capacitive sensors based on parallel plate electrodes can measure shear forces as the effective surface of electrodes changes under parallel forces. In the case of nanocomposite materials, the capacitive effect is due to the gaps between the MWCNTs. The tunneling capacitance between the MWCNTs causes an electric field distribution within the composite [25]. Therefore, if the gaps change by varying the MWCNT concentration or the pressure, the permittivity of the nanocomposite $\epsilon_{r}\left(P\right)$ changes. The following model describes the total characteristic of the sensors in relation to the pressure *P*:

(4) $$C\left(P\right)=\frac{\epsilon_{0}\epsilon_{r}\left(P\right)A}{d}$$

where $\epsilon_{r}\left(P\right)$ is the dielectric constant of the material between the parallel plate electrodes, *A* is the area, and *d* is the distance between the parallel plate electrodes [26].

### 3.3. Impedance Behavior

**Figure 2:**
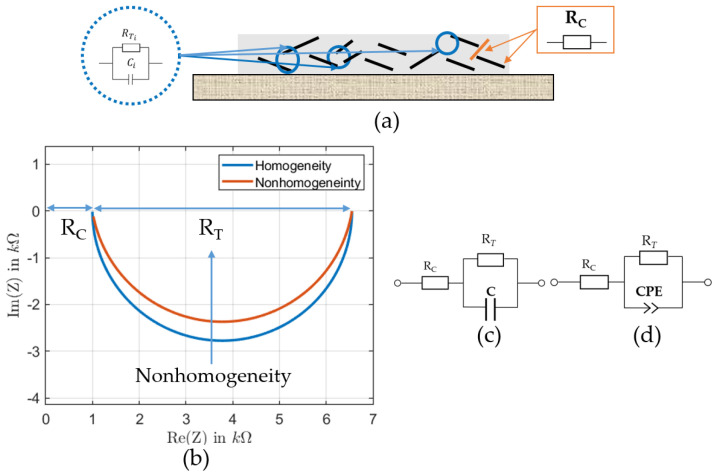
(**a**) Impedance contributions of MWCNTs and MWCNT gaps to the total impedance of the nanocomposite; (**b**) exaggerated behavior of the impedance spectrum of a homogeneous and nonhomogeneous MWCNT nanocomposite; (**c**) equivalent circuit for a homogeneous nanocomposite; (**d**) equivalent circuit for a nonhomogeneous nanocomposite.

For a deep understanding of the principle of the MWCNTs/PDMS-based pressure sensor, resistive and capacitive behaviors should be considered in combination to each other. According to Equation (2), the information about the intrinsic resistance and the intertube of the MWCNTs can be extracted. The resistance can be used to determine the adequate concentration of the nanofiller material at the percolation threshold. The gaps between the MNCNTs can be characterized by the capacitive measurement as shown in Equation (4). As the MWCNTs are randomly distributed they form an *R–C* complex network, which can be characterized by the impedance behavior, which includes the resistive and the capacitive part. Impedance spectroscopy can therefore be used to investigate the complex conduction mechanism within the MWCNTs network. The fitting of impedance spectra to suitable models can provide a deeper understanding of the impedance response of the MWCNTs network in relation to the material properties, MWCNTs concentration, and distribution [22]. Ideally, a nanocomposite can be modeled by a resistor $R_{C}$ in series with a parallel combination of $R_{T}$ and a capacitor *C*. This equivalent circuit is suitable when the carbon nanotubes are uniformly distributed in the nanocomposite. In this model, the Nyquist plot of the impedance spectrum is a semicircle. If the nanocomposite is not homogeneous, the semicircle becomes flat and should be described by a constant phase element (CPE) instead of the capacitor, as shown in Figure 2b. The total impedance can be expressed as:

(5) $$Z=R_{C}+\frac{R_{T}}{1+\omega^{2}R_{T}^{2}C^{2}}-j\frac{\omega R_{T}^{2}C}{1+\omega^{2}R_{T}^{2}C^{2}}$$

where $R_{C}$ represents the contact resistance and nanotubes’ intrinsic resistor, and $R_{T}$ represents the tunneling resistance between the MWCNTs.

A constant phase element is defined by the parameters *C*, the capacity, and α, the *CPE* coefficient. The formula is as follows:

(6) $$Z_{CPE}=\frac{1}{C{\left(j\omega \right)}^{\alpha }}$$

If α = 1, the *CPE* is an ideal capacitor. As homogeneity decreases, the α becomes less than 1.

The total impedance for heterogeneous material can be expressed as:

(7) $$Z=R_{C}+\frac{R_{T}}{1+\omega^{2}R_{T}^{2}Z_{CPE}^{2}}-j\frac{\omega R_{T}^{2}Z_{CPE}}{1+\omega^{2}R_{T}^{2}Z_{CPE}^{2}}$$

## 4. Materials and Methods

### 4.1. Preparation of a Textile-based MWCNTs/PDMS Nanocomposite Pressure Sensor

**Figure 3:**
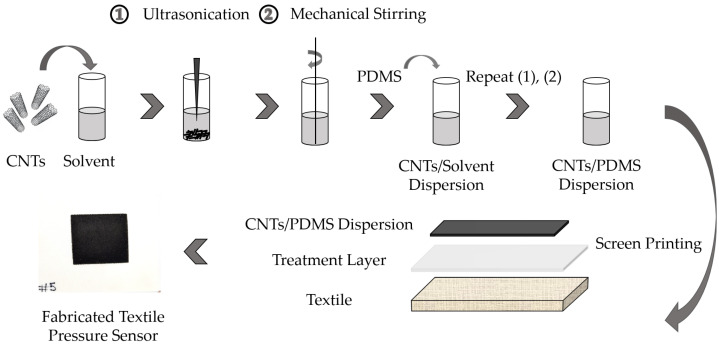
Preparation procedure of textile-based MWCNTs/PDMS nanocomposite pressure sensors.

In order to exploit the potential of the multiwalled carbon nanotubes (MWCNTs) in PDMS, they need to be dispersed homogeneously in the polymer. However, achieving a homogeneous dispersion of MWCNTs in PDMS remains challenging due to the van der Waals attraction force between the MWCNTs, resulting in a reagglomeration of the MWCNTs [27]. Based on our prior experience, tetrahydrofuran (THF) has shown potential for the stable dispersion of MWCNTs in PDMS [25,28]. MWCNTs with an outer diameter of 6–9 nm, an average length of 1 µm, and carbon purity of >95% purchased from Sigma-Aldrich were used as conductive nanofillers, and PDMS (Sylgard 184 Kit) as a base polymer was purchased from Dow Corning. An optimized concentration of 0.3 wt.% of MWCNTs nanocomposite material in PDMS was chosen [25,28]. The dispersion was performed first by a 15-min ultrasound treatment using a Bandelin Sonopuls HD 3200 at an amplitude of 30%, and 15-min magnetic stirring using a CAT M27 at 1500 rpm and a temperature of 70 °C. Subsequently, 5 g of PDMS was added, and the process steps of ultrasonic treatment and magnetic stirring were repeated. In the final step, the hardener was added at a ratio of 10:1 (PDMS/hardener), which was optimum for maintaining the mechanical and electrical properties of PDMS after adding the MWCNTs in it. Once the hardener was added, it needed to be screen printed on the textile substrate within a few hours. A commercial textile was used as a substrate for the sensor. The complete process of the sensor fabrication is shown in Figure 3.

In the curing process, THF should evaporate completely. Due to the formation of bubbles in the nanocomposite thin films when it is subjected to a sudden high temperature environment, initially, the sensor should be cured at room temperature for 120 minutes and then cured at 60 °C for 60 min.

During the screen printing of the nanocomposite material on the textile substrate, various parameters involved in this technique can potentially influence the properties and behavior of the sensor. In this work, a total of 11 samples were produced by varying printing parameters such as squeegee speed, height, and snap-off function when printing, as shown in Table 2. The parameters were as follows: a squeegee speed from 5 to 150 mm/s, a squeegee height from 150 to 350 mm, and a snap-off from 0 to 1 mm. In order to investigate the influence of each parameter, the respective parameter was varied where all other fabrication parameters remained constant. However, in all variations, the thickness of the substrate remained at 0.6 mm and the drying condition was carried out for 10 min at 22 °C and 60 min at 60 °C.

### 4.2. Experimental Setup for Impedance Measurement

**Figure 4:**
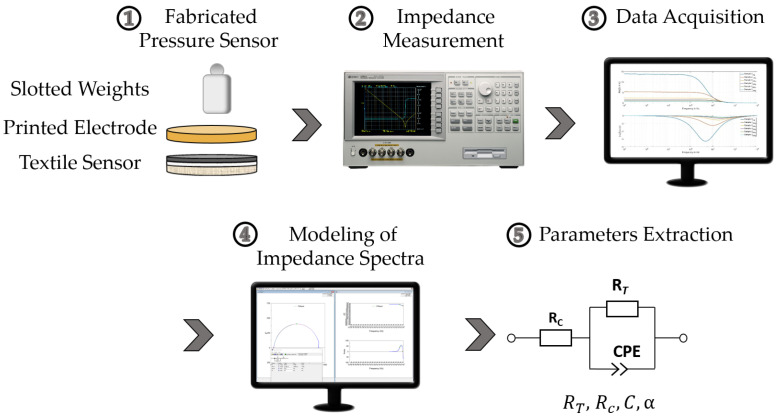
Methodology of the investigation based on impedance spectroscopy.

The impedance measurement setup for the behavioral characterization of the developed pressure sensors is shown in Figure 4. The sample was connected to a precision impedance analyzer (Keysight 4294A), and slotted weights were applied as a load. The impedance analyzer operated in a wide frequency range from 40 Hz to 110 MHz and was configured to measure in R–X mode to obtain the resistive and reactive portion of the sensor’s impedance. Each of the 11 samples was connected to a two-point electrode connected to the impedance analyzer. The sample was successively loaded with weights of 50 g, 100 g, 200 g, 300 g, 400 g, and 500 g. This measurement range corresponded at its high end to the relevant range for wearable sensors. The limit at 50 g was necessary to establish a stable initial contact resistance between electrode and sensitive layer. After each load, 1 minute rest ensured that the sensor returned to its steady state, as it required a response time. Five measurements were carried out for each sample to ensure that the sensor was stabilized. The behavior of the sensor remained stable and reproducible after the third measurement. In this work, the data of the fifth measurement were considered for data optimization.

An equivalent circuit was established to fit the impedance spectra of the selected data using the Zview fitting tool. The equivalent circuit consisted of a resistance in series with a parallel connection of a resistor and a constant phase element (CPE). The resistance $R_{C}$ was a serial resistance resulting from the wires, contact resistance, and nanotubes’ intrinsic resistor. The resistance $R_{T}$ represented the tunnel resistance generated between adjacent and close MWCNTs. Parallel to the tunnel resistance $R_{T}$, a capacity (*C*) was also expected because adjacent MWCNTs acted as the capacitor’s two plates with PDMS as the dielectric. However, the distribution of MWCNTs in PDMS was not ideally homogeneous. It was not considered a pure capacitor, and a constant phase element could represent it. Therefore, the selected model compensated for the system’s inhomogeneities, such as sensor material and wiring. Equation (7) is the model’s total impedance, which has been mentioned in Section 3.3.

## 5. Results and Discussion

### 5.1. Impedance Response under Pressure Loading

In the MWCNTs/PDMS nanocomposite pressure sensor, MWCNTs are distributed in the material matrix, creating conduction paths, and the polymer acts as a tunneling barrier between them. When a load is applied, the sensor material is compressed, the distance between the MWCNTs decreases and the tunneling capacity increases. More MWCNTs move in the direction of the region with a stronger electric field between the underlying interdigitated electrode structures. This behavior can be explained by Equations (2) and (3), where a change in the orientation of a conduction path results in a change in $R_{c}$, and a change of the tunneling distance results in a change in $R_{T}$. The following change in the junction and deformation of MWCNTs increases the overall resistance [29].

**Figure 5:**
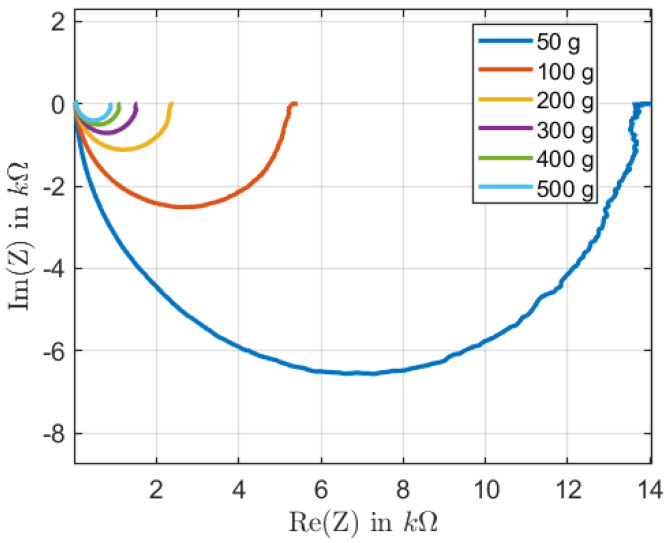
Nyquist plot for sample 2 with weights from 50 g to 500 g.

The impedance measurements showed that the samples exhibited a resistive behavior when measured without an applied weight. The lack of percolation paths explained this behavior. The neighboring MWCNTs were too far apart, resulting in a too-small capacity. As the weight increased, the sample’s impedance exhibited a quasi-semicircular behavior. The semicircle diameter decreased as the sample was exposed to more weight. A significant change in both the capacitive and resistive components occurred due to the structural change of the sample, as shown in Figure 5.

**Figure 6:**
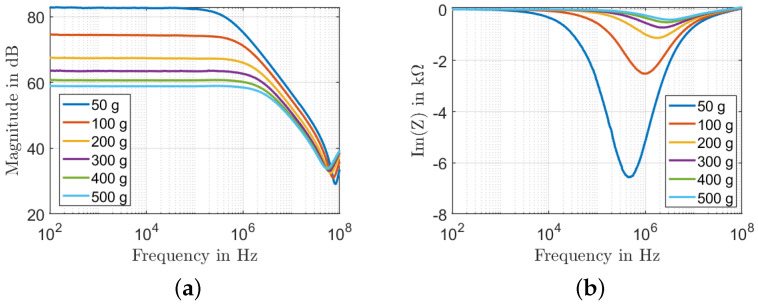
Influence of weights for sample 2: (**a**) Bode plot of the impedance magnitude; (**b**) imaginary part of the impedance as a function of frequency.

Figure 6 shows that the frequency response of the MWCNTs/PDMS nanocomposite pressure sensor depends on the weight ranging from 50 g to 500 g. The nanocomposite sensor exhibits two different behaviors depending on the frequency range: frequency-independent (low frequencies up to the critical frequency, $f_{c}$) and a frequency-dependent response. In the low-frequency range, MWCNTs resistive networks dominate the conduction mechanism, and consequently, the impedance response remains constant until the critical frequency is reached. For instance, while the $f_{c}$ at 50 g load is 3.09 kHz, it shifts to 229.1 kHz at a 500 g load. This can be explained by the charge carriers that flow over capacitive networks at higher frequencies than the critical frequency, proving the dominance of capacitive paths in the conduction mechanism. According to Figure 6a, at low frequencies, the magnitude decreases from 83 dB to 59 dB at 100 Hz, corresponding to 28% of change. The sensor shows a capacitive behavior at the critical frequency, and the impedance amplitude decreases rapidly. Figure 6b shows the change of Im(Z) as a function of the applied frequency. In this case, it can be seen that Im(Z) exhibits two distinct characteristic regions depending on the operating frequency, and the imaginary part Im(Z) increases with an increasing applied load. The sensitivity to load is higher in the low-frequency range up to 100 kHz.

**Figure 7:**
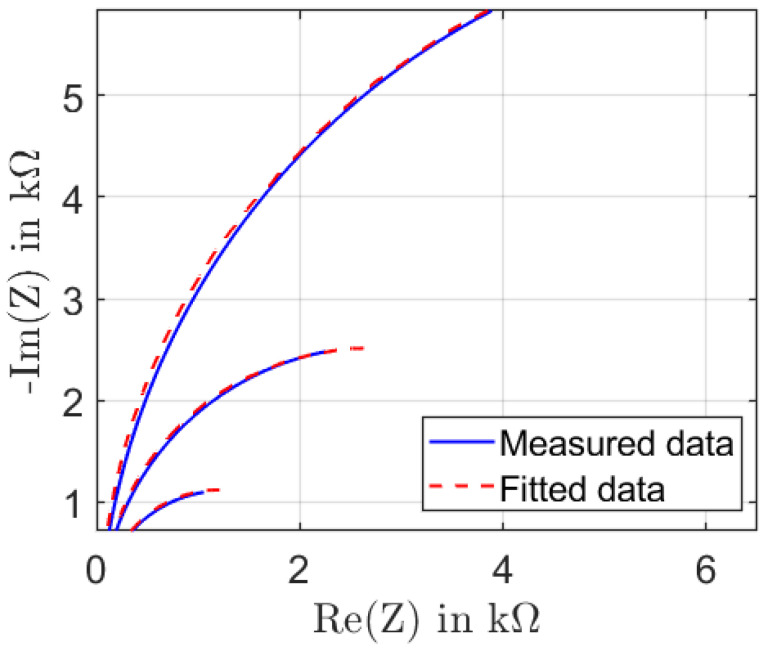
Fitting of the impedance spectra.

In order to extract the equivalent circuit parameters to study the homogeneity of the samples, we fit the measured data to the model in Figure 2d. As shown in Figure 7, the measurement data were noisy at low frequencies due to the limitations of the measurement equipment in this impedance region. Therefore, we decided to fit the model to the half of the impedance spectrum with better data quality. The data were fitted using Zview software.

Figure 7 shows, as an example, the fitting results for sample 2. The impedance changes in the resistive part and imaginary part when an external force is applied to the sample, resulting in a decrease in $R_{T}$ and increased capacity *C* under pressure. In addition, the impedance characteristics of the MWCNTs/PDMS sensor exhibit a resistive behavior in the low-frequency range, while in the high-frequency range, the capacitive behavior dominates. When the MWCNTs/PDMS sample is compressed, the distance between the MWCNTs decreases, and the probability of parallelism of the MWCNTs increases [20] leading to an increase in the surface of the elementary capacitors in the nanocomposite.

### 5.2. Optimization of Manufacturing Parameter

The thickness of the MWCNTs/PDMS material layer realized by screen printing depends on many parameters, especially the squeegee speed, squeegee height, and snap-off distance. The choice of printing parameters is essential to ensure good sensor properties, homogeneity, and sensitivity. The impedance spectra of several sensor samples were fitted to the proposed equivalent circuit in Figure 2d, and the equivalent circuit parameters were extracted for depth to investigate the sensor characteristics.

#### 5.2.1. Influence of Squeegee Speed

In order to investigate the influence of the squeegee speed, several sensors were realized at different squeegee speeds and subjected to an investigation of sensitivity under different pressure loads. In total, five squeegee speeds were considered: 5 mm/s, 10 mm/s, 50 mm/s, 100 mm/s and 150 mm/s and the investigations were carried out under different loads from 50 g to 500 g. A higher squeegee speed results in thicker printed films on the substrate [30] as the nanocomposite does not have enough time to intrude the textile substrate. This is advantageous for the MWCNTs/PDMS pressure sensors, as a higher thickness of the nanocomposite films leads to more MWCNTs networks, resulting in high-pressure sensitivity.

**Figure 8:**
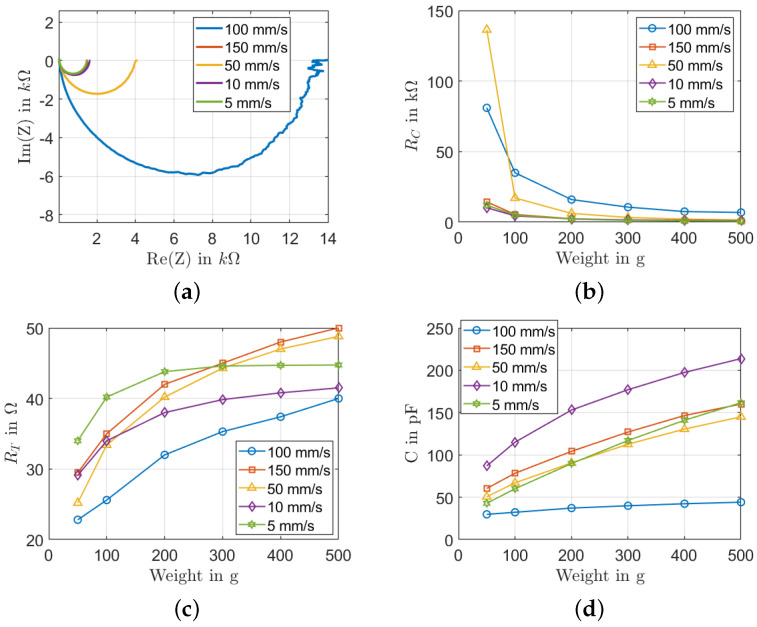
Influence of the squeegee speed: (**a**) Nyquist plot for a load of 300 g; (**b**) $R_{C}$ change with different loads; (**c**) $R_{T}$ change with different loads; (**d**) *C* change with different load.

The Nyquist plot in Figure 8a shows that the real part of impedance increases with increasing pressure, but the imaginary part of the impedance decreases. A big change of 12 kΩ in the real part of impedance between 50 mm/s and 100 mm/s can be observed. For the imaginary part, the change is around 4.4 kΩ. The change is not big for a squeegee speed between 5 mm/s and 50 mm/s. For a squeegee speed of 150 mm/s, the Nyquist plot is near the pressure region of 5 mm/s and 50 mm/s. It may be explained by the optimal region of the squeegee speed for the printing. The $R_{C}$ represents the contact resistances and the intrinsic resistances of the nanotubes in the nanocomposite. Figure 8b shows that the resistance $R_{C}$ decreases with an increasing weight force and increases with an increasing printing speed. The behavior of $R_{C}$ corresponds to the behavior expressed in Equations (2) and (3). At a load of 100 g, the resistance $R_{c}$ increases by 28 kΩ when the squeegee speed increases from 5 mm/s to 100 mm/s. However, the opposite behavior can be seen when the squeegee speed increases from 100 mm/s to 150 mm/s. As explained before, this may be because the optimal value has been reached. Moreover, there is no big change when the squeegee speed is between 5 and 50 mm/s. The decrease of $R_{C}$ is mainly due to the decrease of the contact resistance between the electrode and the sample when a higher weight is applied. Furthermore, this can be explained by the increase in the number of MWCNTs when the speed increases. The resistance change of different samples is not big between 20 kΩ and 80 kΩ, only the sample with a squeegee speed of 50 mm/s at a 50 g load reaches 140 kΩ. In Figure 8c, the resistance change until 200 g is about 10 kΩ for a squeegee speed 100 mm/s and 5 mm/s. When the load is greater than 200 g, the change is small, and a constant plateau is observed for the parameter $R_{T}$ after 100 g, for a squeegee speed of 5 mm/s, for example. This can be explained by the bulkiness of PDMS introduced in thicker samples that resist the movement of MWCNTs networks in the PDMS. Finally, the capacitance varies from 30 to 210 pF, as illustrated in Figure 8d. The capacitance of the sensor increases as the load increases. As the sensor gets compressed when the load is applied, the distance between the MWCNTs decreases, thereby increasing the tunneling capacitance as expressed by Equation (4). Moreover, more MWCNTs are moved towards the stronger fields between the underlying electrode structures, increasing the material’s dielectric constant. For both reasons, the capacitance increases when the load increases.

#### 5.2.2. Influence of Squeegee Height

**Figure 9:**
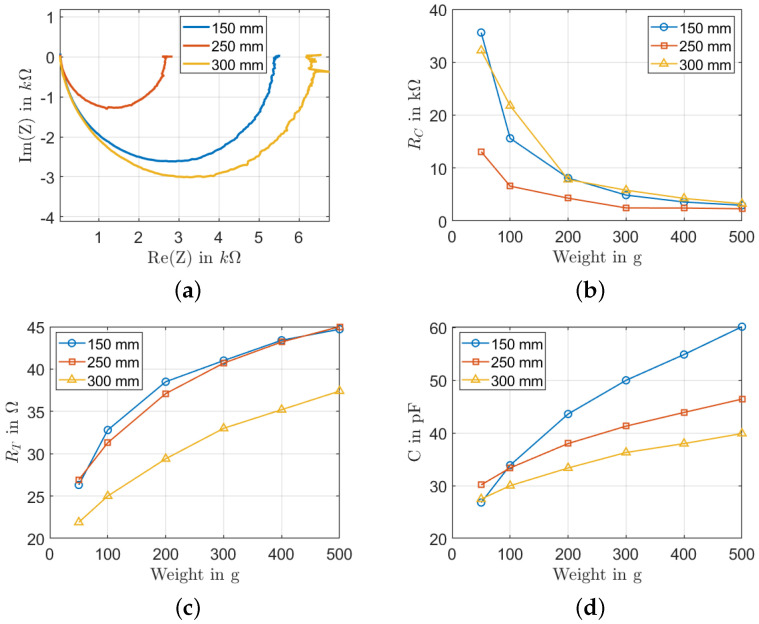
Influence of the squeegee height: (**a**) Nyquist plot at a load of 300 g; (**b**) $R_{C}$ change with different loads; (**c**) $R_{T}$ change with different loads; (**d**) *C* change with different load.

Three squeegee heights were tested for screen printing: 150 mm, 250 mm, and 300 mm. In Figure 9a, the Nyquist plot shows that the the sample printed with a squeegee height of 300 mm has the widest semicircle. In Figure 9b, the $R_{C}$ exhibits a constant behavior for loads higher than 200 g for all the samples with a resistance of around 5 kΩ. This behavior can be explained by the improved contact between the electrode and the sensor when the load increases. For the resistance $R_{T}$ in Figure 9c, both samples with squeegee heights of 150 mm and 250 mm have similar values. The sample with a squeegee height of 300 mm exhibits a lower value of $R_{T}$, indicating the existence of longer percolation paths between MWCNTs in the film, contributing to the total impedance in series to each other. The tunneling capacitance decreases when the height increases indicating the existence of more elementary capacitances along the percolation paths between MWCNTs, contributing to the total impedance in series to each other. Both the tunneling resistance $R_{T}$ and the tunneling capacitance $C_{T}$ increase when the acting force increases. In the case of interdigitated electrodes, the acting forces let more MWCNTs move towards the region with a stronger field between the interdigitated electrodes. This is why both *C* and $R_{T}$ increase with the increase of the applied weight.

#### 5.2.3. Influence of the Snap-Off Distance

**Figure 10:**
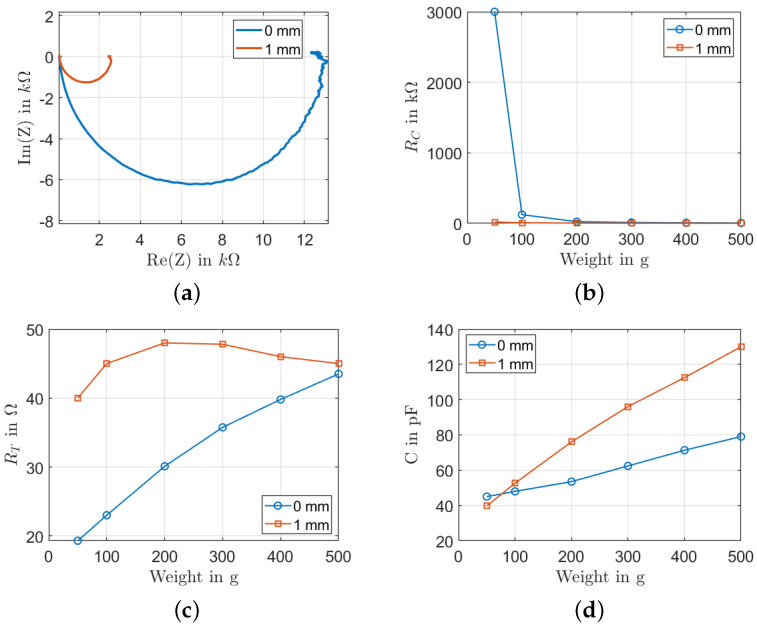
Influence of the snap-off distance: (**a**) Nyquist plot at a load of 300 g; (**b**) $R_{C}$ change with different loads; (**c**) $R_{T}$ change with different loads; (**d**) *C* change with different load.

The introduction of a buckling reduces the distance between the screen and the substrate. This leads to the formation of a nonhomogeneous surface of the printed films. To investigate this effect, two different snap-off distances, 0 mm and 1 mm, were tested (Figure 10). The snap-off distance is the distance between the substrate and the bottom side of the screen. Figure 10a shows that the impedance of the sample with a snap-off distance of 1 mm is much smaller than that of the sample with a 0 mm snap-off distance. The $R_{C}$ is almost constant, independent from the acting weight, as shown in Figure 10b. For the sample with a snap-off distance of 0 mm, only at a low load value of 50 g is the $R_{C}$ big with a value of 300 kΩ, mainly due to the bad electrode contact. The resistance $R_{T}$ increases when the snap-off distance increases (Figure 10c). When the load is 50 g, the resistance $R_{T}$ is equal to 19 kΩ and 40 kΩ when the snap-off distance is 0 mm and 1 mm, respectively. As the load increases, the curves converge, and the $R_{T}$ is equal to 45 kΩ for a load of 500 g. However, the opposite behavior for the capacitance is shown in Figure 10d. Both samples start with around 40 pF at a load of 50 g and increase but diverge as the load increases. This increase is explained by the decrease in the distances between MWCNTs.

### 5.3. Manufacturing Anomalies

During printing, several effects may influence the sensor’s quality, especially in the case of a textile substrate. Therefore, it is essential to have a nondestructive method for characterizing the sensors and detecting production anomalies. With such a method, the production’s success can be monitored during the quality control, and the sensors can be sorted into good and bad after production. These problems can be related to the homogeneity of the nanocomposite material and the interface between the nanocomposite and the textile.

#### 5.3.1. Morphological Characterization of Nonhomogenity

**Figure 11:**
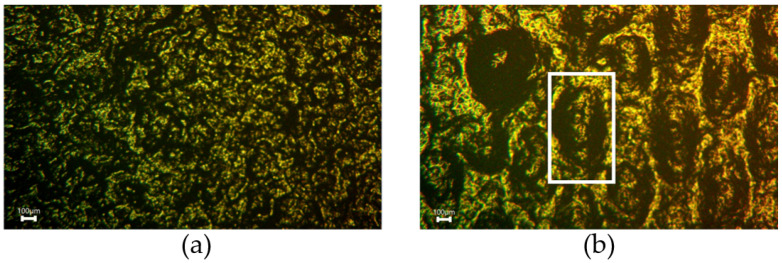
Microscopic investigations for different printed sensors: (**a**) homogeneous sample, (**b**) sample having bubbles.

In order to visualize the quality of the MWCNTs/PDMS nanocomposite in the fabric and the surface’s homogeneity, the samples were investigated by microscope imaging. Some examples are given in Figure 11, where the morphology of two sensors realized with different printing parameters are shown. In Figure 11a, the microscopic image of sensor S3 is shown, which was fabricated with the following printing parameters: squeegee speed of 50 mm/s, squeegee height of 200 mm, and snap-off distance of 0 mm. The realized MWCNTs/PDMS film is homogeneous. Figure 11b illustrates the microscopic image of the sensor S11, which was fabricated with the following parameters: squeegee speed of 100 mm/s, squeegee height of 200 mm, and snap-off distance of 1mm. The deposited layer is, in this case, not homogeneous, and bubbles are visible. That can be explained by the high printing speed as the squeegee speed affects the amount of material flowing into the mesh of the openings in the fabric. The faster the speed, the fewer materials penetrate the fabric. Furthermore, the snap-off distance is critical in screen printing. When the distance between the squeegee and the textile is low, more pressure is applied, and a thin layer of material can be deposited in the substrate. The latter ensures a minimum air gap and fewer air bubbles on the surface.

Based on the model parameters of the impedance spectra, nonhomogeneity can be characterized by the parameter α of the *CPE* (Equation (6)) as is explained in Figure 2. When α = 1, the *CPE* becomes a pure capacitor, meaning that the material is homogeneous and the distance between the MWCNTs is overall the same. For samples S3 and S11, α was equal to 0.96 and 0.95, respectively. The parameter α is very sensitive to homogeneity. In our tests, the parameter α had a small deviation from the value 1. This indicated a distribution of the distances between the MWCNTs in the composite and, therefore, more nonhomogeneity. The smaller this parameter is, the more nonhomogeneity exists. This is confirmed by the theory related to the *CPE* and is shown, e.g., in Figure 11. This is why this parameter has a good potential for characterizing the sensor fabrication quality. Furthermore, the parameter α is the same for a certain sample under different loads, which confirms that this parameter is purely related to describing the heterogeneity of the sensor.

#### 5.3.2. Influence of Manufacturing Parameters on Homogeneity

After elaborating the hypothesis, we used the parameter α to characterize the quality of the sensor in this section, which compares the parameter α considering all screen-printed sensors.

**Figure 12:**
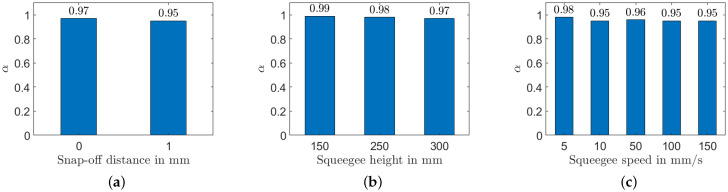
Influence of different screen-printing parameters on the CPE coefficient α: (**a**) snap-off distance; (**b**) squeegee height; (**c**) squeegee speed.

As shown in Figure 12a, two snap-off distances were compared: 0 mm and 1 mm. The sample had an α equal to 0.97 when the snap-off equaled 0 mm. This indicated a better homogeneity, which could be explained by a reduced distance between the substrate and the squeegee resulting in more pressure on the substrate. When the snap-off distance was increased, the penetration of nanocomposite material inside the textile substrate was not homogeneous, which resulted in a nonuniform surface of thin films and a nonhomogeneous distribution of MWCNTs in PDMS. Four different heights were considered for the squeegee height: 150 mm, 250 mm, and 300 mm, as shown in Figure 12b. The parameter α decreased, so using a squeegee at a short height was better for a more homogeneous sample. Finally, the α of the squeegee speed was compared. Five different speeds were considered: 5 mm/s, 10 mm/s, 50 mm/s, 100 mm/s, and 150 mm/s, as illustrated in Figure 12c. The α changes decreased from 0.98 to 0.95, when the squeegee speed increased. When the squeegee speed was high, the MWCNTs/PDMS material did not have enough time to penetrate perfectly into the textile’s fiber matrix. Furthermore, the high speed resulted in the formation of air bubbles in the deposited layer of the MWCNTs/PDMS. Choosing a low snap-off distance, small squeegee height, and low squeegee speed is therefore suggested to fabricate a high-quality textile pressure sensor with good homogeneity. Finally, implementing the parameter α in the production process is a valid method as the last step for quality checks.

## 6. Conclusions

Textile pressure sensors have attracted more interest as they can be used in various applications. Printing in textiles is still challenging as they require more treatments. Furthermore, different screen-printing parameters highly influence sensor fabrication. In this work, we fabricated MWCNTs/PDMS nanocomposite pressure sensors with different printing parameters such as squeegee speed, squeegee height, and snap-off distance. These sensors were characterized by impedance spectroscopy for a wide range of frequencies from 40 Hz to 110 MHz. The sensor provided a quasi-semicircle response, which could be modeled as a simplified $R_{C}$-$R_{T}$//*CPE*. Then, the extracted fitting parameters were used to investigate the influence of parameters in the screen-printing technique on the fabricated sensor. It could be seen that when the applied load increased, $R_{C}$ decreased and $R_{T}$ and $C_{T}$ increased. When the printing speed increased, it was observed that the $R_{C}$ increased because of the increase of the number of MWCNTs with the increase in speed. In addition, the sample with a squeegee height of 300 mm showed the lowest value of $R_{T}$, because of longer percolation paths. Indeed, the extracted parameters supported the investigation of manufacturing anomalies, such as the thickness of the nanocomposite thin films and the nonhomogeneity of the MWCNT distribution in PDMS. Based on the results, the potential of the parameter α can be exploited to investigate the sensor material homogeneity, which is influenced by various parameters in the material synthesis and the screen-printing parameters. A small snap-off distance, small squeegee height, and low squeegee speed are suggested. 

## Figures and Tables

**Table 1:** Characterization methods for nanocomposite sensors.

	Ref.	Characterization Method	Sensor Type	Material Composition	Material Concentration	Substrate
90Microcharacterization methods	[13]	Scanning electron microscopy (SEM)	Cotton flexible capacitive pressure sensor	Thermoplastic polyurethane (TPU) composite	7 wt.% TPU	Carbonized cotton fabric
	[14]	Raman spectroscopy	3D piezoresistive sensor	MWCNT-NWs	0.1 wt.% MWCNTs	Nonwoven (NW) fabric
	[15]	Atomic force microscopy (AFM)	Electrochemical flexible biosensor	Gold (Au), molybdenum disulfide nanoparticles (Mo$S_{2}$ NPs), and Au	-	Polyethylene terephthalate (PET)
	[16]	Transmission electron microscopy (TEM)	Capacitive pressure sensor	PDMS/MWCNT	1, 2, 4, 5, and 6 wt.%	Polyethylene terephthalate (PET)
90Electrical methods	[17]	Resistance measurement	Flexible pressure sensor	PDMS/MWCNT	0.1 wt.%	PDMS
	[18]	van der Pauw method	Reduced GO-based cotton electrode	Reduced graphene oxide-cotton (rGOC)	4 mgmL${}^{-1}$ of GO dispersion in water	Cotton fabric
	[2]	Capacitance measurement	Capacitive sensor	Carbon black (CB)/silicone rubber (SR) composite	0, 4, 6, 8, and 10 wt.%	Textile
	[22]	Impedance spectroscopy	Piezoresistive strain sensor	MWCNTs/epoxy nanocomposite	1 wt.%	Glass-reinforced epoxy laminate substrate (FR4)

**Table 2:** Printing parameters for the pressure sensor fabrication.

Sample	Squeegee Speed	Squeegee Pressure	Squeegee Height	Snap-Off
	(mm/s)	(N)	(mm)	(mm)
S1	100	160	200	0
S2	150			
S3	50			
S4	10			
S5	5			
S6	100		150	
S7			250	
S8			300	
S9		8	350	
S10		0	200	
S11				1

## Data Availability

The data supporting this study’s findings are available from the corresponding author, upon reasonable request.
